# Assessing the relation between fatigue and depression in multiples sclerosis: the impact of measurement choice

**DOI:** 10.3389/fimmu.2026.1838616

**Published:** 2026-07-15

**Authors:** Tino Zaehle, Stefanie Linnhoff

**Affiliations:** 1 Institute for Medical Psychology, Otto-von-Guericke University, Magdeburg, Magdeburg, Germany; 2 Department of Neurology, Otto-von-Guericke University, Magdeburg, Germany

**Keywords:** BDI-FS, BDI-II, depression, fatigue, multiple sclerosis (MS)

## Abstract

**Background:**

Fatigue and depression are among the most prevalent and disabling symptoms in multiple sclerosis (MS) and are frequently reported to be strongly associated. This association is often interpreted as reflecting shared pathophysiology. However, widely used depression scales such as the Beck Depression Inventory-II (BDI-II) include somatic items that overlap with core features of MS and MS-related fatigue, potentially inflating observed relationships.

**Objectives:**

To examine whether the association between depression and fatigue in MS depends on the choice of depression measure, we compared the BDI-II with the Beck Depression Inventory–Fast Screen (BDI-FS), a subscale designed to minimize somatic confounding.

**Methods:**

In a cohort of 86 people with MS, self-reported fatigue (MFIS, WEIMuS) and depressive symptoms (BDI-II, BDI-FS) were assessed. Fatigue scores were standardized to allow comparability across instruments. Pearson correlations were computed to quantify associations between depression and fatigue, and dependent correlations were compared.

**Results:**

Depressive symptoms assessed with the BDI-II showed strong positive correlations with fatigue (*r* = .60 -.66, all *p* <.001). In contrast, BDI-FS scores showed significantly weaker, moderate associations (all *r* ≤.43, *p* <.001). Direct comparisons confirmed that correlations between BDI-II and fatigue were significantly stronger than those between BDI-FS and fatigue (all *p* <.001).

**Conclusions:**

The strength of the association between depression and fatigue in MS depends substantially on the depression measure used. The commonly reported strong relationship appears to be inflated by somatic item overlap in traditional depression scales. The BDI-FS provides a more specific assessment of depressive symptoms and may improve diagnostic accuracy and the interpretation of symptom relationships in MS.

## Introduction

Fatigue is one of the most prevalent and disabling symptoms of multiple sclerosis (MS), affecting up to two-thirds of patients and substantially impairing daily functioning and quality of life (1–3). Depression is likewise highly prevalent in people with MS, occurring at rates two- to three-fold higher than in the general population, and frequently co-occurs with fatigue and other symptoms such as cognitive slowing, pain, and sleep disturbance (4–6). Accordingly, numerous studies report moderate to strong associations between fatigue and depressive symptomatology in MS (3, 7, 8). This prominent association has led many authors to propose shared mechanistic pathways and has, in clinical practice, contributed to considerable diagnostic uncertainty, where fatigue may be misinterpreted as depression, or depressive symptoms may be obscured by severe fatigue.

However, neurobiological evidence suggests a more differentiated relationship. Structural and functional imaging studies indicate that fatigue and depression in MS are associated with partially overlapping but largely distinct neural networks. Depression has primarily been linked to alterations within cortico-limbic circuits, whereas fatigue has been associated with disruptions in cortico-thalamo-basal ganglia and attentional networks, with overlap emerging mainly in regions involved in affective and attentional integration such as the cingulate cortex (9). Longitudinal findings further support a bidirectional but non-identical relationship, with depressive symptoms predicting later fatigue and persistent fatigue increasing the risk of subsequent depression (10). Together, these data suggest a bidirectional but non-identical relationship, with distinct etiological components.

Against this background, a critical methodological issue has received comparatively little attention. Widely used depression scales, including the Beck Depression Inventory-II (BDI-II, 11), contain several somatic items, such as low energy, sleep disturbance, concentration difficulties, and reduced activity level that overlap directly with core symptoms of MS and MS-related fatigue (12). This problem has been highlighted in psychometric work on depression assessment in MS, including the “trunk-and-branch” model, which distinguishes symptoms more specific to depression from symptoms that may also reflect MS-related disease burden (13). Similarly, systematic reviews and head-to-head comparisons of depression measures in MS have shown that instrument choice and symptom content can substantially affect the interpretation of depressive symptom scores (12, 14). As a result, people with MS may obtain elevated scores on these items due to disease-related symptom burden rather than affective depression. This overlap can inflate correlations between depression and fatigue measures and may contribute to the consistently strong associations reported in the literature (12).

This issue is particularly relevant in fatigue research, where it is often necessary to distinguish between fatigue and depression or to exclude patients with clinically relevant depressive symptoms. If depression is assessed using instruments that include fatigue-related items, fatigue-dominant patients may be misclassified as depressed, leading to biased sample selection and potentially distorted conclusions.

One approach to addressing this problem is the use of depression measures that minimize somatic content. The Beck Depression Inventory – Fast Screen (BDI-FS) is a short form of the BDI-II that focuses on cognitive-affective symptoms and was specifically developed for use in populations with medical or neurological illness (11). Previous work in MS has shown that the BDI-FS correlates with external indicators of mood disturbance while demonstrating reduced associations with fatigue and neurological disability, suggesting improved construct specificity in this population (12, 15, 16).

The present study provides a direct empirical test of whether the observed association between depression and fatigue in MS depends on the choice of depression measure. In a cohort of people with MS, we compared correlations between fatigue severity and depressive symptoms assessed using the standard BDI-II and the BDI-FS. While previous work has examined the psychometric properties of the BDI-FS in MS, the present study extends this literature by directly comparing depression–fatigue associations obtained from the BDI-II and the BDI-FS within the same cohort. Demonstrating that the choice of depression measure fundamentally alters the observed relationship would argue strongly for integrating non-somatic scales such as the BDI-FS into both clinical practice and research, thereby improving diagnostic clarity and the interpretability of symptom correlations in MS populations.

Based on prior evidence, we hypothesized that (I) depressive symptoms measured with the BDI-II would show strong positive correlations with fatigue, reflecting both true associations and symptom overlap, whereas (II) depressive symptoms measured with the BDI-FS would show significantly weaker associations with fatigue, consistent with reduced somatic confounding.

## Methods

### Participants

The study sample consisted of 86 people with a confirmed diagnosis of MS (see **Table 1** for demographic and clinical characteristics). Diagnosis of MS was established according to the McDonald criteria (17), and people with MS were required to be at least three months free from clinical relapse or corticosteroid treatment at the time of testing. Participants were excluded if they had any current psychiatric condition, had taken antidepressants, corticosteroids, neuroleptics, or medications targeting fatigue within the preceding three months, or had experienced an acute neurological event in the same period. All participants were on stable disease-modifying therapy.

**Table 1:** Group characteristics, mean (± SD).

Characteristics	MS (N = 86)
Gender [female/male]	64 / 22
Age [years]	42.85 (12.17)
EDSS [points]	2.84 (1.66)
MS duration [years]	10.53 (7.91)
MS form [RRMS / SPMS / PPMS]	81 / 4 / 1
BDI-II [points]	12.70 (7.80)
BDI-FS [points]	2.92 (2.55)
Fatigue total score (z-stand.)	-0.19 (1.03)
Fatigue cognitive score (z-stand.)	-0.03 (0.95)
Fatigue physical score (z-stand.)	-0.32 (1.05)

BDI, Becks Depression Inventory; BDI-FS, Becks Depression Inventory - Fast Screen; EDSS, Expanded Disability Status Scale; MS, Multiple Sclerosis; PPMS, Primary Progressive Multiple Sclerosis; RRMS, Relapsing-Remitting Multiple Sclerosis; SPMS, Secondary Progressive Multiple Sclerosis.

We report data from a consecutive convenience sample of people with MS whose data were acquired between 2022 and 2024 across several outpatient experimental protocols. All participants completed standardized assessments of depressive symptoms using the Beck Depression Inventory-II (BDI-II). Subjective fatigue was assessed using either the Modified Fatigue Impact Scale (MFIS) or the Wuerzburg Fatigue Inventory for Multiple Sclerosis (WEIMuS), depending on the respective study protocol.

The study protocol was approved by the Ethics Committee of the University of Magdeburg. All participants gave written informed consent in accordance with the Declaration of Helsinki.

### Questionnaires

Depressive symptoms were assessed using the Beck Depression Inventory-II (BDI-II, 11). The BDI-II is a 21-item self-report questionnaire widely used to measure the severity of depressive symptoms, including cognitive-affective and somatic domains. The BDI- Fast Screen (BDI-FS, 16) is a 7-item short form of the BDI-II comprising the items sadness, pessimism, past failure, loss of pleasure, self-dislike, self-criticalness, and suicidal thoughts or wishes. It focuses exclusively on cognitive-affective symptoms and was developed to minimize confounding by somatic symptoms in populations with medical illness.

Fatigue severity was assessed using the Modified Fatigue Impact Scale (MFIS, 18) and the Wuerzburger Fatigue Inventory for Multiple Sclerosis (WEIMuS, 19). The MFIS is a 21-item self-report measure assessing the impact of fatigue on physical, cognitive, and psychosocial functioning. The WEIMuS is a validated MS-specific questionnaire consisting of 17 items, capturing both physical and cognitive dimensions of fatigue.

### Statistical analysis

Analyses were conducted using R (version 4.2.0; R Core Team). To allow comparability across fatigue instruments, MFIS and WEIMuS total and subscale scores were standardized (z-scores) based on published normative values (20, 21). Depression was assessed using the BDI-II total score and the BDI-FS score. Pearson correlations were computed to quantify associations between the depression measures and fatigue z-scores. Subsequently, Meng’s Z test was applied to compare dependent correlation coefficients (22).

## Results

### Participant characteristics

Demographic and clinical characteristics of the participants are summarized in **Table 1**. MFIS and WEIMuS scores indicated a moderate to high level of fatigue across the sample, consistent with epidemiological data on MS-related fatigue. Mean depressive symptom severity was in the mild range for both the BDI-II and BDI-FS, although individual scores showed substantial inter-individual variability.

No significant associations were observed between demographic variables (age, sex) or EDSS and MS duration and either depression measure (all |r| < 0.18, p > 0.12), allowing us to evaluate depression–fatigue associations without adjustment for these covariates in initial analyses.

### Correlation between BDI-II/BDI-FS and fatigue measures

**Figure 1:**
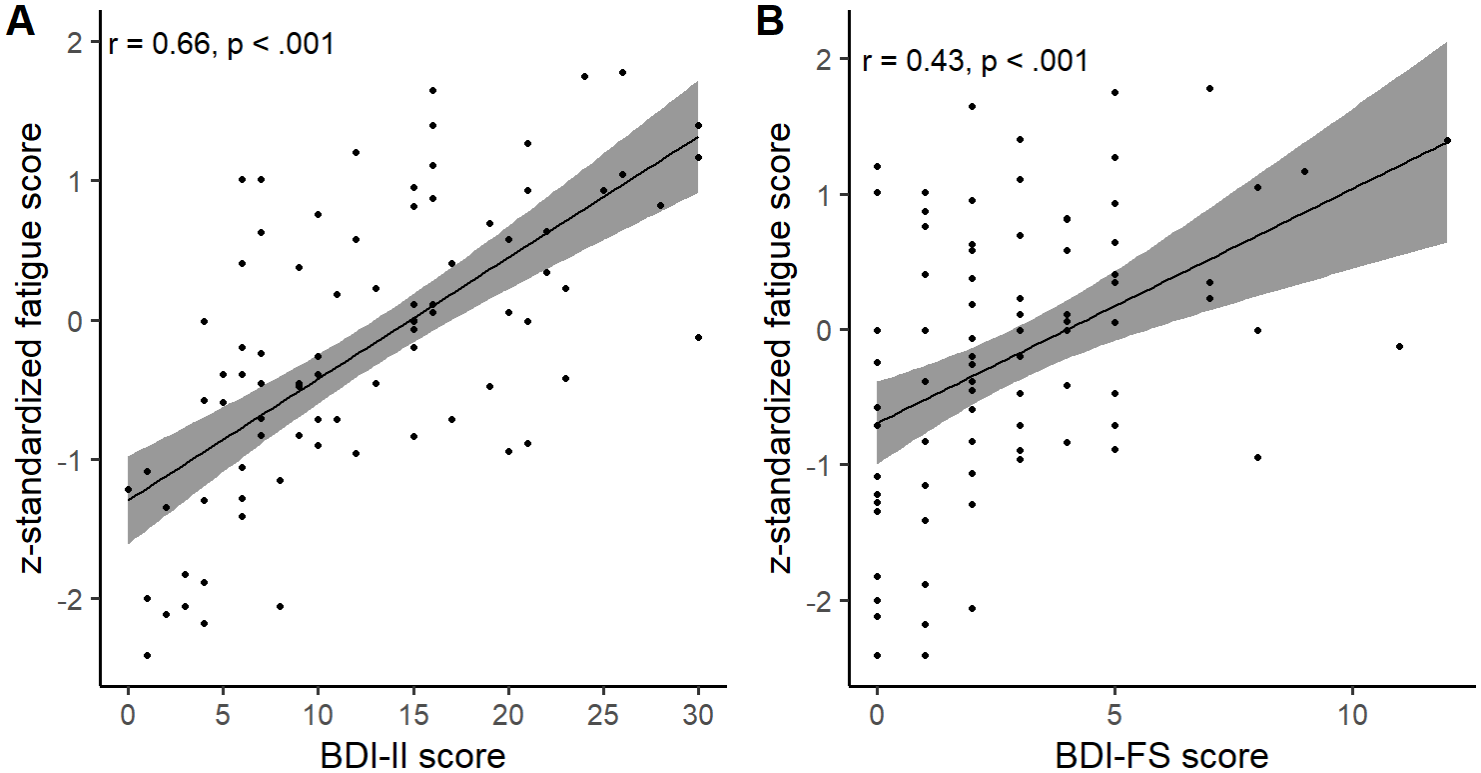
Influence of depression measure on the association between depressive scores and fatigue in multiple sclerosis (n = 86). **(A)** The BDI-II total score showed a strong positive correlation with z-standardized fatigue severity. **(B)** The BDI-FS showed a markedly weaker association with z-standardized fatigue severity. Fatigue scores were z-standardized across MFIS and WEIMuS scores using published normative values. Dots represent individual participants; lines indicate linear regression with 95% confidence intervals. Pearson correlation coefficients are shown in each panel.

BDI-II scores showed strong positive correlations with both fatigue scores (**Figure 1A**). The association with the fatigue total score was robust (*r* = .663, *p* <.001), driven by significant correlations with both the physical (*r* = .601, *p* <.001) and cognitive subscales (*r* = .644, *p* <.001).

**Figure 2:**
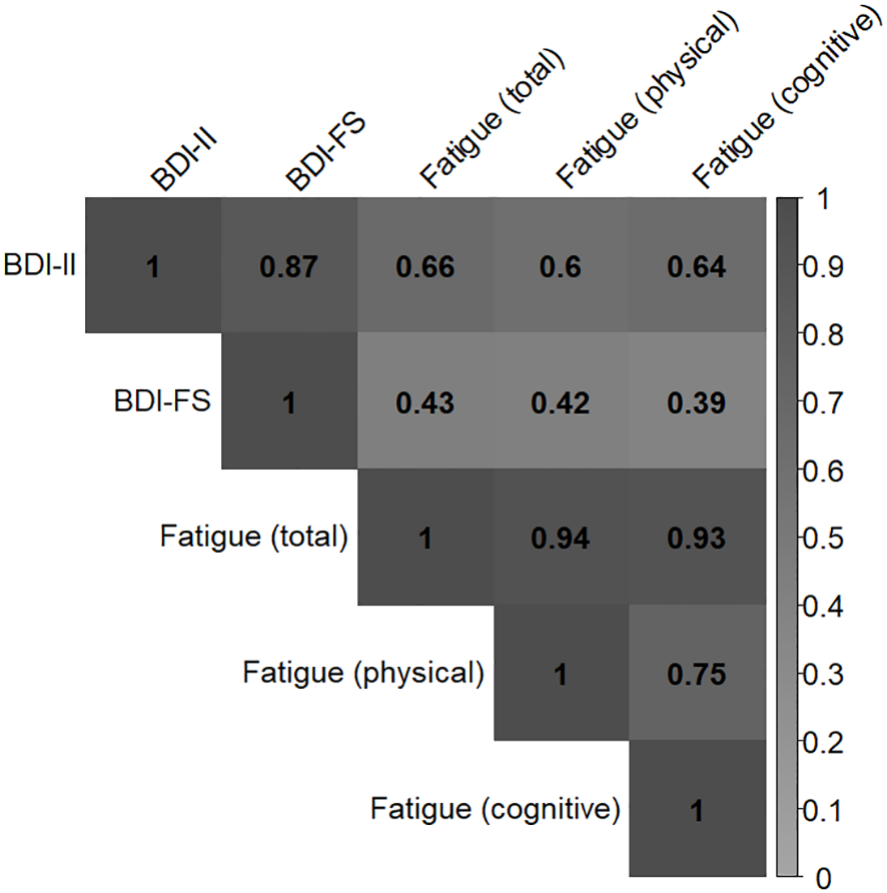
Correlation matrix of depression measures and fatigue dimensions in multiple sclerosis (n = 86). The heat map displays Pearson correlation coefficients between depression measures (BDI-II total and BDI-FS score) and z-standardized fatigue scores (total, physical, and cognitive). Fatigue scores were z-standardized across MFIS and WEIMuS scores using published normative values. Color intensity reflects the strength of positive correlations, with darker shades indicating stronger associations. Correlation coefficients (r) are shown within each cell.

BDI-FS scores showed weaker, but significant correlation with fatigue severity (**Figure 1B**). The association with the fatigue total score was medium (*r* = .430, *p* <.001), driven by significant correlations with both the physical (*r* = .415, *p* <.001) and cognitive subscales (*r* = .387, *p* <.001). **Figure 2** shows the correlation matrix between depression measures and fatigue dimensions. For all fatigue scores, the correlations between BDI-II and fatigue scores were significantly stronger than the correlations between BDI-FS and fatigue scores (z > 3.8 , p < .001; Meng et al., 1992) (cf. **Table 2**).

**Table 2:** Meng-Z-test (r(BDI-II total / BDI-FS: .866), n = 86).

Fatigue score	r (fatigue/BDI-II total)	r (fatigue/BDI-FS)	z	*p* (two-sided)
fatigue total score	.663	.430	5.01	< .001
fatigue physical score	.601	.415	3.85	< .001
fatigue cognitive score	.644	.387	5.38	< .001

Additional partial correlation analyses controlling for age, sex, EDSS, and disease duration yielded virtually identical results. The overall pattern of associations remained unchanged, with BDI-FS scores showing weaker correlations with fatigue than the BDI-II score.

## Discussion

In the present study, we directly compared the association between depressive symptoms and fatigue in MS using two psychometric instruments: the BDI-II and the BDI-FS, the latter specifically designed to isolate cognitive–affective symptoms of depression. Consistent with prior literature, depressive symptoms assessed with BDI-II showed strong positive correlations with self-reported fatigue. In contrast, depressive symptoms assessed with the BDI-FS exhibited moderate and statistically significant weaker associations with subjective fatigue measures. These findings support the view that the frequently reported relationship between fatigue and depression in MS is, at least in part, driven by measurement artefacts, particularly the inclusion of somatic items that overlap with core features of MS-related fatigue.

This interpretation is consistent with earlier clinical work showing that fatigue and depression are both highly relevant for quality of life in MS, but do not necessarily represent the same construct. For example, Janardhan and Bakshi (23) demonstrated that fatigue and depression independently contribute to impaired quality of life, while broader reviews have emphasized the clinical burden and diagnostic complexity of both symptom domains in MS (6, 24, 25).

Our results extend earlier validation work demonstrating that the BDI-FS functions as a psychometrically more specific depression screener in medically ill populations. Benedict et al. (15) showed that the BDI-FS correlates with external indicators of depressed mood while remaining largely insensitive to neurological disability and fatigue. Likewise, Hind et al. (12) highlighted that commonly used depression scales such as the BDI-II, CES-D, and PHQ-9 are susceptible to somatic contamination in MS, whereas the BDI-FS largely avoids this limitation. This concern is also consistent with the “trunk-and-branch” model proposed by Strober and Arnett (13), which distinguishes symptoms that may reflect MS-related disease burden from symptoms more specific to depression. In addition, head-to-head comparisons of depression measures in MS have shown that commonly used self-report instruments are not interchangeable and that instrument choice can influence the identification of clinically relevant depressive symptoms (14).

The co-occurrence of fatigue and depression in MS has often been interpreted as reflecting shared pathophysiological mechanisms (3, 8). However, neurobiological and symptom-level evidence suggests a more nuanced picture. Fatigue has primarily been linked to disruptions in cortico-thalamo-basal ganglia and attentional networks, whereas depression is more strongly associated with cortico-limbic circuitry (9). Complementing these neurobiological findings, recent symptom-network analyses in early relapsing-remitting MS showed that fatigue is closely connected to depressive symptoms, but that this relationship is not reducible to a single overlapping symptom such as tiredness (26). Longitudinal studies further suggest bidirectional but non-identical relationships between fatigue and depression (10). Our findings help reconcile these observations. When somatic symptom overlap is minimized, the association between depression and fatigue is substantially reduced, suggesting that previously reported strong correlations may partly reflect item-level overlap rather than a fully shared underlying mechanism.

From a clinical perspective, these findings support the use of the BDI-FS as a more appropriate depression screener in MS. Its brevity enhances feasibility in both clinical and research settings, while its focus on cognitive–affective symptoms improves diagnostic specificity. Importantly, patients with pronounced fatigue may be misclassified as depressed when assessed with somatically loaded instruments such as the BDI-II (12). Such misclassification has practical consequences, including inappropriate treatment decisions and the misinterpretation of fatigue as an affective disorder.

Beyond clinical assessment, the choice of depression measure has important implications for research. Using instruments that minimize somatic confounding allows for a more accurate estimation of the independent and interactive contributions of fatigue and depression. This is particularly relevant in neuroimaging studies, where somatic contamination may lead to spurious associations between depression scores and neural systems primarily related to fatigue or disability (9). Similarly, in clinical trials targeting fatigue or depression, the use of non-somatic depression measures may reduce bias in estimating treatment effects.

### Limitations and future directions

Several limitations should be considered. First, detailed MS-related clinical variables, including time since the last relapse, MRI disease activity, and current or previous disease-modifying treatments, were not systematically available for all participants. These factors may influence both fatigue and depressive symptoms and should be incorporated in future studies. Second, participants with diagnosed depression or antidepressant treatment were excluded, which limits generalizability to the broader MS population. This criterion was chosen to reduce psychiatric confounding and to allow a more focused examination of how depression measurement influences fatigue–depression associations in individuals without diagnosed depression. Nevertheless, some individuals showed elevated BDI-II scores, consistent with the fact that self-report measures assess symptom severity on a continuum and may capture subclinical symptoms as well as MS-related somatic complaints. The modest sample size should also be considered when interpreting the findings, and replication in larger and more clinically heterogeneous MS cohorts is warranted. Third, the BDI-FS is a screening instrument rather than a diagnostic tool; therefore, clinical interviews remain necessary for diagnostic confirmation, and optimal cut-off scores in MS require further validation. Finally, future studies using item-level analyses may clarify which specific BDI-II symptoms contribute most strongly to fatigue–depression associations in MS and whether somatic symptom items show stronger associations with fatigue than cognitive–affective items.

## Conclusion

By directly contrasting depression–fatigue associations obtained using the BDI-II and the BDI-FS within the same individuals and formally evaluating the correlations using Meng’s Z test for dependent correlations, the present study demonstrates that the commonly reported strong relationship between fatigue and depression in MS is partly driven by somatic symptom overlap. When depression is assessed using a measure that excludes somatic content, its association with fatigue is markedly reduced. These findings highlight the importance of appropriate measurement selection and support the BDI-FS as a clinically and methodologically preferable instrument for assessing depression in MS.

## Data Availability

The raw data supporting the conclusions of this article will be made available by the authors, without undue reservation.
